# DEMENTIA CARE REIMAGINED: THE MULTIMODAL PREVENTING LOSS OF INDEPENDENCE THROUGH EXERCISE (PLIÉ) PROGRAM

**DOI:** 10.1093/geroni/igac059.679

**Published:** 2022-12-20

**Authors:** Deborah Barnes, Francesca Nicosia, Theresa Allison

## Abstract

Over 6 million people in the United States and 50 million people worldwide are living with some form of dementia. Current pharmacotherapies for people living with dementia (PLWD) can provide some symptomatic relief but do not alter the disease course, have numerous side effects, and do not improve quality of life. Non-pharmacologic and behavioral interventions are increasingly recognized as inexpensive and effective ways to improve a wide range of outcomes for PLWD. Yet few interventions target multiple domains related to dementia quality of life. This symposium overviews the Preventing Loss of Independence through Exercise (PLIÉ) program, an evidence-based, integrative mind-body group movement program that targets key domains associated with better quality of life in PLWD: physical function, cognitive function, well-being, social connection, and self-esteem. This symposium provides an overview of the broad impact of this novel, non-pharmacologic, multimodal intervention on quality-of-life outcomes for PLWD, including expansion to include care partners and people with mild cognitive impairment and implementation in varied settings including adult day programs, nursing homes, and online delivery. It includes a participatory component for attendees to experience PLIÉ in action. Discussion focuses on the need for programs that support dementia quality of life through engagement in mind-body movement and social connection.

